# Association of *APOE* With Primary Open-Angle Glaucoma Suggests a Protective Effect for *APOE ɛ4*

**DOI:** 10.1167/iovs.61.8.3

**Published:** 2020-07-02

**Authors:** Milica A. Margeta, Sophia M. Letcher, Robert P. Igo, Jessica N. Cooke Bailey, Louis R. Pasquale, Jonathan L. Haines, Oleg Butovsky, Janey L. Wiggs, for the NEIGHBORHOOD consortium

**Affiliations:** 1 Department of Ophthalmology, Massachusetts Eye and Ear Infirmary and Harvard Medical School, Boston, Massachusetts, United States; 2 Department of Population and Quantitative Health Sciences, Case Western Reserve University School of Medicine, Cleveland, Ohio, United States; 3 Cleveland Institute for Computational Biology, Case Western Reserve University, Cleveland, Ohio, United States; 4 Department of Ophthalmology, Icahn School of Medicine at Mt. Sinai, New York, New York, United States; 5 Ann Romney Center for Neurologic Diseases, Department of Neurology, Brigham and Women's Hospital, Harvard Medical School, Boston, Massachusetts, United States; 6 Evergrande Center for Immunologic Diseases, Brigham and Women's Hospital, Harvard Medical School, Boston, Massachusetts, United States

**Keywords:** glaucoma, genetics, microglia, APOE, TREM2

## Abstract

**Purpose:**

Prior studies have demonstrated that microglial activation is involved in the pathogenesis of primary open-angle glaucoma (POAG). Here we sought to identify genetic associations between POAG and variants in *APOE* and *TREM2,* genes associated with Alzheimer disease (AD) that critically regulate microglial neurodegeneration-associated molecular signature.

**Methods:**

*APOE* genotypes were called using imputed data from the NEIGHBOR consortium (2120 POAG cases, 2262 controls) and a second cohort from the Massachusetts Eye and Ear Infirmary (MEEI; 486 cases, 344 controls). *TREM2* coding variants were genotyped by means of the Illumina HumanExome BeadArray. The data set was analyzed for association with POAG overall, as well as the high-tension glaucoma (HTG) and normal-tension glaucoma (NTG) subgroups, using logistic regression adjusting for age and sex.

**Results:**

In the combined NEIGHBOR-MEEI data set, significant association was observed for *APOE ε4* in POAG overall (odds ratio [OR], 0.83; 95% confidence interval [CI], 0.74–0.94; *P* = 0.0022) and in both the HTG subgroup (OR, 0.81; 95% CI, 0.70–0.94; *P* = 0.0052) and NTG subgroup (OR, 0.71; 95% CI, 0.58–0.87; *P* = 0.0014). A rare *TREM2* variant (A105V) was found only in HTG cases (3 of 2863 cases) and in none of the controls (*P* = 0.03). Three *TREM2* rare variants associated with AD were not significantly associated with POAG (*P* > 0.05).

**Conclusions:**

We have found that the *APOE ε4* allele is associated with a reduced risk of POAG. Interestingly, the same allele is adversely associated with AD, suggesting a mechanistic difference between neurodegenerative diseases of the eye and the brain. *TREM2* variants associated with AD did not significantly contribute to POAG risk.

Primary open-angle glaucoma (POAG) is a genetically and clinically heterogeneous neurodegenerative disease whose main hallmark is retinal ganglion cell (RGC) apoptosis. Glaucoma pathogenesis remains poorly understood, and there are currently no clinically approved therapies that directly promote RGC survival.^1^ Prior research has demonstrated that in terms of genetic risk, there exists overlap between glaucoma and neurodegenerative diseases of the brain. For example, some of the same genes that harbor known risk alleles for amyotrophic lateral sclerosis (ALS), such as optineurin (*OPTN*), TANK-binding kinase 1 (*TBK1*), and ataxin2 (*ATXN2*), have also been shown to confer genetic risk in glaucoma.^2^^–^^8^ Herein we sought to explore parallels between glaucoma and another common age-related neurodegenerative disease, Alzheimer disease (AD), which is characterized by accumulation of amyloid-β plaques, neurofibrillary tangles, neuronal loss, and inflammation.^9^^,^^10^

The strongest risk factor for late-onset AD is apolipoprotein E (*APOE*), the major lipoprotein in the brain. *APOE* has three variants in humans (*ε2, ε3*, and *ε4*), which differ by two amino acid residues. The ε3 allele is the most common and is considered the baseline for AD risk; *ε4* raises risk of AD relative to ε3, whereas *ε2* is protective.^11^^,^^12^ More recently, rare variants of the transmembrane receptor *TREM2* (triggering receptor expressed on myeloid cells 2) have also been identified as risk factors for AD^13^^–^^16^ and Nasu-Hakola disease, a neurodegenerative disease characterized by early dementia and bone cysts with fractures.^17^ Interestingly, in the brain, *TREM2* is only expressed by myeloid cells (resident microglia and peripherally derived monocytes/macrophages),^18^ highlighting the importance of this cell type in the pathogenesis of AD.^19^^–^^22^ While *APOE* is expressed more broadly, recent work has found that *APOE* is upregulated in microglia in mouse models of neurodegenerative disease, including AD, ALS, and multiple sclerosis.^23^^–^^25^ Furthermore, *TREM2* and *APOE* have been identified as key regulators of the microglial molecular phenotype associated with neurodegeneration (the so-called microglial neurodegenerative or disease-associated microglia molecular signature).^23^^–^^25^ Given that microglia have also been implicated in the pathogenesis of glaucoma^26^^–^^30^ and that *APOE* is upregulated in the retina and the aqueous humor of patients with glaucoma,^31^^,^^32^ we hypothesized that *APOE* and *TREM2* may have genetic associations with POAG as well.

The genetic association between *APOE* and POAG has been previously examined in a series of small and likely underpowered studies that identified conflicting results.^33^^–^^42^ Several meta-analyses found no association between *APOE* and glaucoma,^43^^,^^44^ while others reported an association between the *APOE ε4/ε4* genotype and POAG in Asians.^45^^,^^46^ Another large meta-analysis reported an association between a single-nucleotide polymorphism (SNP) in the promoter region of *APOE* (rs449647) and glaucoma^47^; this SNP, however, is not associated with AD.^48^ Interestingly, *APOE ε4* is associated with decreased risk for another common neurodegenerative disease of the eye, age-related macular degeneration (AMD),^49^^–^^52^ opposite of its effect on AD. To our knowledge, the genetic association between *TREM2* and POAG has not been previously explored.

In this study, we sought to examine the associations between *APOE* and *TREM2* with POAG in a relatively large data set that includes subgroups with high-tension glaucoma (HTG) and normal-tension glaucoma (NTG). We find that *APOE ε4* is associated with reduced risk of POAG, especially NTG. Three *TREM2* rare variants associated with AD were not significantly associated with POAG, while a rare *TREM2* variant (not implicated in AD) may contribute to HTG risk.

## Methods

### Study Participants

This study adhered to the tenets of the Declaration of Helsinki and has been reviewed and approved by the Institutional Review Boards of the Massachusetts Eye and Ear Infirmary, Harvard School of Public Health, the Brigham and Women's Hospital, University of Pittsburgh, Johns Hopkins University, Duke University, University of West Virginia, University of Miami, University of Michigan, Stanford University, Marshfield Clinic, and the University of California, San Diego. Informed consent was obtained from the participants after explanation of the nature and possible consequences of the study.

Two case-control genome-wide association study (GWAS) data sets were used for this study: the Mass Eye and Ear Infirmary (MEEI) component of the Glaucoma Genes and Environment (GLAUGEN) GWAS^53^ and the National Eye Institute Glaucoma Human Genetics Collaboration (NEIGHBOR) GWAS.^54^ Detailed information on these data sets has been described previously.^4^^,^^53^^,^^54^ Briefly, the MEEI component of the GLAUGEN data set includes 486 POAG cases and 344 controls, and the NEIGHBOR data set includes 2120 POAG cases and 2262 controls. Average age of enrollment was 64.5 ± 11.0 years for MEEI GLAUGEN controls and 62.0 ± 11.2 years for the MEEI GLAUGEN cases, as well as 68.9 ± 11.4 years for the NEIGHBOR controls and 66.6 ± 13.7 years for the NEIGHBOR cases. The MEEI GLAUGEN cases and controls were 58.4% and 59.8% female, respectively, while the NEIGHBOR cases and controls were 54.1% and 56.5% female, respectively.

A harmonized definition of POAG was adopted across these data sets based on the following criteria: (1) open anterior segment angles, (2) reproducible glaucomatous visual field loss on reliable tests or (3) an eye with cup-disc ratio of at least 0.7 with one visual field showing glaucomatous loss, and (4) no identifiable secondary cause for optic nerve disease. Elevated intraocular pressure (IOP) was not a criterion for POAG definition, but if present, there had to be no secondary causes on anterior segment examination.

Sixty-seven percent of cases had a history of elevated IOP (≥22 mm Hg) measured in a clinical setting (typically between the hours of 8:00 am and 5:00 pm) and were classified as HTG. Cases with IOP <22 mm Hg (without treatment) measured in the clinic at the time of study enrollment were classified as NTG. Cases undergoing IOP-lowering therapy at the time of enrollment were included in the HTG group if they had a documented history of IOP >22 mm Hg prior to treatment, and cases undergoing IOP-lowering therapy at the time of enrollment were included in the NTG group if they did not have recorded pressures >22 mm Hg before treatment. Pretreatment IOP measurements were not available for all cases.

### Genotyping

The MEEI and NEIGHBOR case control data sets were originally genotyped as part of the GLAUGEN and NEIGHBOR GWAS studies as previously described.^4^^,^^53^^,^^54^ Subsequently, the genotype data were imputed to the Haplotype Reference Consortium panel^55^ using the Michigan Imputation Server.^56^ *APOE* genotypes (including alleles *ε2, ε3*, and *ε4*) were determined from haplotypes of rs429358 and rs7412 (T-T, T-C, and C-C, respectively), the two SNPs known to define *APOE* alleles. Imputation scores were high for both SNPs (*r*^2^ > 0.93). Haplotypes are unambiguous from the unphased genotypes except for the double heterozygote, rs429358 C/T-rs7412 C/T, which was called *ε2/ε4* rather than as *ε1/ε3*, since the *ε1* allele is extremely rare.

Fourteen *TREM2* rare variants (minor allele frequency [MAF] <1%, call rate ≥98%) were extracted from Illumina HumanExome BeadArray (Illumina, Inc., San Diego, CA) genotype data for the NEIGHBORHOOD and GLAUGEN data sets. We focused on *TREM2* rare variants as those have previously been associated with AD.^13^^–^^15^ Genotyping was completed at the Center for Inherited Disease Research. The Illumina Genome Studio (Illumina) and PLINK^57^ were used for all quality controls (QC) steps except where noted. Basic QC for samples included screens for call rate (≥98.5%) and high (≥95%) concordance with a previous Illumina 660K panel run on the same sample^54^ where available (about 80% of samples). We verified recorded sex in the clinical records with genotyped sex by two criteria: mean fluorescence intensity on the X and Y chromosomes, plus genotype heterozygosity on the X chromosome and call rate on the Y, allowing male and female samples to have heterozygous X-linked and successful Y-linked genotypes, respectively. We tested samples for pairwise relationships and unexpected duplication using KING.^58^

We verified European ancestry from the first two principal components derived from genotypes at 9000 ancestry-informative markers by means of the SNPweights program,^59^ including representative HapMap CEU, YRI, CHB, and JPT samples as reference populations. Moreover, we conducted a principal components analysis over 52,040 independent (pairwise *r*^2^ < 0.1), common (MAF ≥0.005) SNPs using the smartpca program in EIGENSOFT^60^ to detect finer population structure. Of the first 20 principal components, the first, sixth, and eighth were significantly associated (*P* < 0.05 by logistic regression) with POAG status.

Initial QC screens for markers included call rate (≥98%) and consistency with Hardy-Weinberg proportions (*P* > 10^−6^ by Fisher exact test). We screened markers for differences in allele frequency between whole-genome-amplified DNA samples and all other samples by the Fisher exact test and removed from analysis all markers with *P* < 0.0001. All pseudoautosomal, Y-linked, and mitochondrial SNPs were subject to review in Illumina Genome Studio (Illumina, Inc.) and, if necessary, were reclustered by hand. We also confirmed genotype clustering for rare (MAF <0.02) variants from fluorescence intensity data by means of zCall, run with a stringent *z* score threshold of *z* = 21 for calling heterozygous genotypes. Every rare SNP with two or more additional heterozygous calls by zCall than by GenCall was reviewed in Genome Studio, and if necessary, cluster locations were adjusted manually.

### Analyses

#### *APOE* Alleles.

Each of the three detectable *APOE* alleles was tested for association with glaucoma using logistic regression with age and sex as covariates. Results for *ε2* and *ε4* were compared to *ε3* as the reference. A likelihood ratio test (LRT) was run to compare the logistic regression models that include age and sex and with or without the number of *ε2* and *ε4* alleles to assess the overall significance of adding *APOE* genotypes. *APOE* genotype frequencies were compared among POAG, HTG, and NTG cases and controls using the Pearson χ^2^ test.

#### *TREM2* Rare Variants

Association between individual variants and POAG case/control status was assessed using logistic regression, including as covariates age at exam, sex, and three principal components observed to be significantly associated with POAG.

## Results

**Figure 1.**
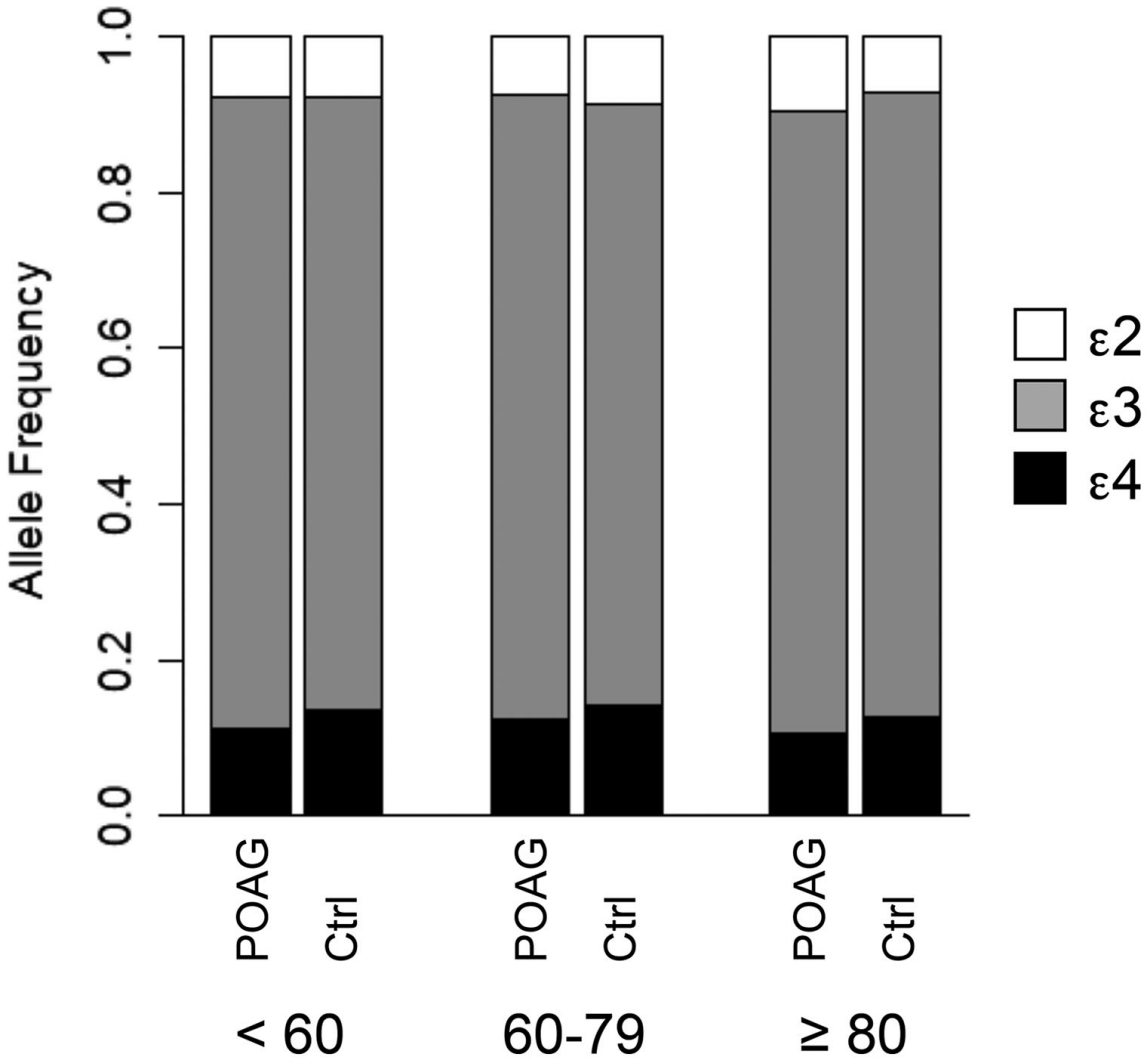
Distribution of *APOE* alleles according to age. Allele frequencies were not significantly different across age groups in either cases or controls (*P* > 0.2 from each of six pairwise comparisons).

In the combined NEIGHBOR-MEEI data set, *APOE ε4* was inversely associated with POAG overall (odds ratio [OR], 0.83; 95% confidence interval [CI], 0.74–0.94; *P* = 0.0022) and in both the HTG (OR, 0.81; 95% CI, 0.70–0.94; *P* = 0.0052) and NTG (OR, 0.71; 95% CI, 0.58–0.87; *P* = 0.0014) subgroups (Table 1). The LRT, considering all alleles jointly, confirmed these findings and demonstrated the most significant difference in overall allele frequencies with NTG (*P* = 0.0041). The *APOE* allele frequencies did not significantly differ among POAG cases and controls when grouped according to age (Fig. 1).

**Table 1.** *APOE* Allelic Association With POAG, HTG, and NTG

	NEIGHBOR					
Allele	POAG(2120 Cases, 2262 Controls)		HTG(978 Cases, 2262 Controls)		NTG(395 Cases, 2262 Controls)	
	OR (95% CI)	*P* Value	OR (95% CI)	*P* Value	OR (95% CI)	*P* Value
*ε2*	0.96 (0.83–1.13)	0.65	0.94 (0.77–1.15)	0.57	1.09 (0.83–1.42)	0.52
*ε4*	0.84 (0.74–0.96)	0.0078	0.81 (0.68–0.97)	0.013	0.69 (0.54–0.88)	0.0036
LRT		0.021		0.03		0.0099
	**MEEI**					
	POAG(486 Cases, 344 Controls)		HTG(320 Cases, 344 Controls)		NTG(166 Cases, 344 Controls)	
	OR (95% CI)	*P* Value	OR (95% CI)	*P* Value	OR (95% CI)	*P* Value
*ε2*	1.08 (0.74–1.60)	0.68	1.18 (0.77–1.79)	0.45	0.94 (0.54–1.59)	0.82
*ε4*	0.81 (0.60–1.10)	0.18	0.81 (0.58–1.13)	0.22	0.81 (0.53–1.20)	0.30
LRT		0.39		0.38		0.55
	**Combined data set**					
	POAG(2606 Cases, 2606 Controls)		HTG(1298 Cases, 2606 Controls)		NTG(561 Cases, 2606 Controls)	
	OR (95% CI)	*P* Value	OR (95% CI)	*P* Value	OR (95% CI)	*P* Value
*ε2*	0.97 (0.84–1.12)	0.70	0.98 (0.81–1.17)	0.80	1.01 (0.80–1.28)	0.92
*ε4*	0.83 (0.74–0.94)	0.0022	0.81 (0.70–0.94)	0.0052	0.71 (0.58–0.87)	0.0014
LRT		0.0067		0.02		0.0041

**Figure 2.**
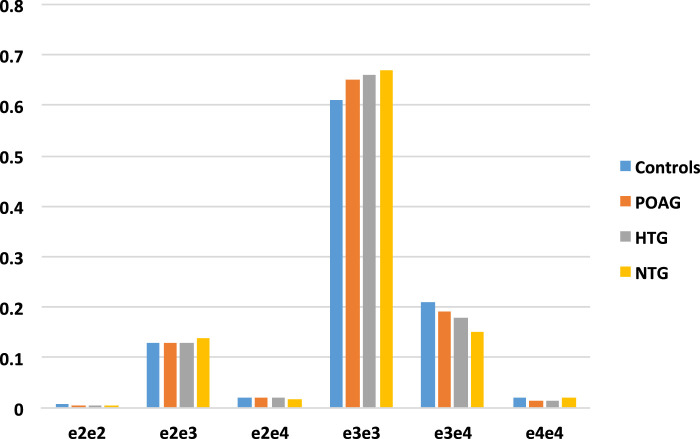
*APOE* genotype distribution among POAG, HTG, and NTG cases and controls. *P* values for distribution compared to controls are POAG, 0.02; HTG, 0.085; and NTG, 0.008.

The distribution of *APOE* genotypes among POAG overall, HTG, and NTG cases and controls differed significantly, with the largest effect observed for the NTG case versus control comparison (*P* = 0.008; POAG, *P* = 0.02 and HTG, *P* = 0.085) (Fig. 2).

To determine whether the *ε4* allele is associated with delayed age of disease onset, we compared the mean age of diagnosis for the *ε3ε3* POAG cases with the mean age of diagnosis for the *ε3ε4* POAG cases. While the mean (65.8 vs. 64.9 years) and median (67.3 vs. 65.6 years) ages of onset were indeed greater in the *e3/e4* cases than in *e3/e3* cases, the difference was not significant (*P* = 0.14 by *t* test, *P* = 0.13 by Wilcoxon rank-sum test), likely due to limited sample size (463 *e3/e4* POAG cases).

*TREM2* is known to interact with *APOE*, and rare *TREM2* coding variants have been associated with AD.^13^^–^^15^ To determine whether any rare *TREM2* coding variants are associated with POAG, we extracted NEIGHBOR and MEEI case-control association data for variants genotyped using the Human Exome bead array. Seven rare (MAF <1%) *TREM2* missense variants were identified, and one variant (A105V) was identified in three HTG cases and in no controls (*P* = 0.03) (Table 2). Comparing the HTG allele frequency to the European Caucasian population in population database the Genome Aggregation Database v.2.1.1 (gnomAD)^67^ provided support for enrichment in HTG cases (*P* = 0.008). The remaining *TREM2* variants, including variants known to contribute to AD risk, did not demonstrate comparable enrichment (*P* > 0.05).

**Table 2.** *TREM2* Rare Variants From the Human Exome Array

rsID	Variant	CADD	PP	SIFT	gnomAD(MAF)	Controls(MAF)	POAG(MAF)	HTG(MAF)	NTG(MAF)	*P* *Case/Control	*P* ^†^GnomAD
rs200392967	D39E	23.4	PS	D	1.3E-4	1/6381 (1.5E-4)	0/5628 (0.00)	0/2866 (0.00)	0/1158 (0.00)	0.99	0.99
rs143332484	R62H^‡^	11.11	B	T	0.012	69/6347 (0.011)	49/5623 (0.009)	30/2836 (0.011)	10/1148 (0.009)	0.24	0.06
rs142232675	D87N^‡^	22.8	PD	T	0.002	4/6412 (6.2E-4)	4/5668 (7.1E-4)	0/2866 (0.00)	1/1157 (8.6E-4)	0.86	0.008
rs145080901	A105V	24.2	PD	D	1.3E-4	0/6416 (0.00)	3/5669 (5.3E-4)	3/2863 (1.0E-3)	0/1158 (0.00)	0.07 (POAG)0.03 (HTG)	0.05(POAG)0.008(HTG)
rs149622783	R136Q	1.841	B	T	1.3E-4	2/6414 (3.1E-4)	3/5669 (5.3E-4)	1/2865 (3.5E-4)	1/1157 (8.6E-4)	0.56	0.05
rs79011726	E151K	23.2	B	T	1.8E-4	3/6413 (4.7E-4)	1/5671 (1.8E-4)	0/2866 (0.00)	0/1158 (0.00)	0.29	0.99
rs2234255	H157Y^‡^	23.1	PS	D	2.9E-4	3/6413 (4.7E-4)	2/5670 (3.5E-4)	0/2866 (0.00)	1/1157 (8.6E-4)	0.76	0.67

B, benign; CADD, combined annotation-dependent depletion score; D, damaging; PD, probably damaging; PP, polyphen2; PS, possibly damaging; SIFT, sorting intolerant from tolerant score; T, tolerated.

* *P* value for logistic regression using cases and controls. Cases are POAG overall except for A105V, where the results are provided for both POAG overall and HTG.

† Comparison of POAG cases to the European population distribution in GnomAD using the Fisher exact test. For A105V, the results are provided for both POAG and HTG.

‡ Previously associated with AD.^13^^–^^16^

## Discussion

In this study, we have examined the genetic association of POAG with *APOE* and *TREM2*, two well-established risk factors for AD. We selected the *APOE ε2, ε3,* and *ε4* alleles for evaluation because of the known and important contributions of these alleles to risk for other neurodegenerative diseases, specifically AD^11^^,^^12^ and age-related macular degeneration.^49^^–^^51^ Interestingly, we have found that *APOE ε4*, which is positively associated with AD, is inversely associated with POAG. This effect was most pronounced in NTG, consistent with *APOE* playing a direct role in regulating RGC degeneration rather than indirectly via IOP regulation. Although prior published reports on the association of *APOE* and glaucoma had small sample sizes and reported conflicting results, it is worth noting that two studies that also demonstrated a protective effect for *APOE ε4* in glaucoma were larger than the others (with more than 300 enrolled participants) and also enriched in patients with NTG.^33^^,^^34^ Lam et al.^34^ demonstrated that *APOE ε4* was protective in NTG patients of Chinese ancestry, while Mabuchi et al.^33^ found that *ε4* was inversely associated with open-angle glaucoma in Japan, where the predominant form of glaucoma is the NTG subtype.^61^ We speculate that the strength of association noted between *APOE ε4* and POAG will depend on the proportion of NTG patients enrolled in a given study, which may explain some of the variability in the published individual studies^33^^–^^42^ and meta-analyses.^43^^–^^47^

What is the mechanism by which *APOE ε4* may be protective in glaucoma? *APOE* is expressed in a variety of cell types in the healthy retina and the optic nerve, including Müller glia and astrocytes.^62^ However, *APOE* is also upregulated in the neurodegeneration-associated microglia in the brain in a variety of neurodegenerative disease mouse models, including AD, ALS, and multiple sclerosis.^23^^,^^24^ Furthermore, *APOE* has been found to be a critical regulator of this microglial neurodegenerative phenotype that develops in response to apoptotic neurons.^23^ We speculate that *APOE* may be similarly upregulated in microglia in glaucoma, as microglial reactivity has been found to contribute to glaucoma pathogenesis.^26^^–^^30^

Another interesting parallel can be made between our findings and the association of *APOE* with AMD, where *APOE ε4* has also been found to be inversely associated with disease.^49^^–^^52^ The reason why the same *APOE* allele has an opposing relationship in ocular neurodegenerative diseases and AD is presently poorly understood. A study by Levy et al.^63^ has shown that in a mouse model of AMD, mice with human *APOE ε4* allele had lower levels of Monocyte chemoattractant protein-1 (MCP-1/CCL2) (a major monocyte attractant), less myeloid cell accumulation in the subretinal space, and decreased photoreceptor degeneration. Therefore, microglia with the *APOE ε4* allele appear to be less reactive, which may be helpful in retinal neurodegenerations but harmful in AD, which is characterized by toxic Aβ plaques and tau deposits that need to be contained by the immune system. Furthermore, there could be additional mechanisms by which *APOE* plays a role in AD pathogenesis that are unrelated to its effect on the microglial transcriptional phenotype. For instance, APOE stimulates production of the Aβ precursor Amyloid precursor protein (APP) and thus directly contributes to Aβ plaque formation, with *ε4* allele being most potent at producing this effect.^64^

We also investigated the association between glaucoma and *TREM2*, a transmembrane receptor expressed by myeloid cells that has recently been associated with AD.^20^^–^^22^ We have found that rare variants previously associated with AD interrogated by our exome chip (R62H,^13^^,^^14^ D87N,^15^ and H157Y^14^^,^^16^) were not associated with POAG, which further underscores different underlying pathogenic mechanisms in glaucoma and AD. Notably, unlike *APOE ε4, TREM2* rare variants were also not associated with NTG. Although both *APOE* and *TREM2* have been implicated in the regulation of the microglial neurodegeneration-associated phenotype, single-cell RNAseq analysis in a mouse model of AD has found that *TREM2* and *APOE* may regulate different subpopulations of neurodegeneration-associated microglia.^24^ Whether *TREM2* plays a role in regulating microglial molecular signature in the retina will merit further investigation.

An unexpected finding of our study was the association of one rare variant of *TREM2*, A105V, with HTG, although the number of cases was quite small. This variant has a high pathogenicity score based on in silico analyses, is not associated with AD or Nasu-Hakola disease, and is located in a different region of the TREM2 receptor than the AD-associated rare variants. Given the association with HTG only, we speculate that *TREM2* may be involved in IOP regulation. In addition to microglia, *TREM2* is also expressed by peripheral myeloid cells (monocytes and macrophages).^18^ Notably, macrophages are abundantly present in the conventional outflow pathway^65^ and have been implicated in IOP regulation after selective laser trabeculoplasty.^66^ Further research will be necessary to validate our genetic findings and explore the possible role of *TREM2* in IOP regulation.

There were several limitations of our study. First, we do not have whole-exome sequencing data for *TREM2*, and therefore additional *TREM2* rare variants may be associated with glaucoma that were not present on our chip. Second, although our data set is the largest of its kind, its statistical power for association of rare variants is limited. Finally, while we did show a consistent effect for *APOE ε4* association with POAG in two independent data sets (NEIGHBOR and MEEI), additional data sets with a sufficient number of NTG cases are currently not available for further replication.

In summary, our results demonstrate, for the first time, that *APOE ε4* association in glaucoma is strongest in the NTG subgroup and that the *APOE ε4* allele is inversely associated with NTG and also with POAG overall. This result helps clarify the overall contribution of *APOE* to glaucoma risk and suggests that prior conflicting results could have reflected varying numbers of NTG cases in tested cohorts. Understanding the underlying mechanism by which *APOE ε4* is involved in the pathogenesis of glaucoma will necessitate further study in animal glaucoma models (for instance, in *APOE ε2, ε3*, and *ε4* humanized mice, which are commercially available). Interestingly, *TREM2* rare variants associated with AD did not contribute to POAG risk in our study. The association between a different rare variant of *TREM2,* A105V, with HTG is intriguing but at this point preliminary and warrants further study and validation in larger data sets.
